# Molecular mechanisms of atlastin-mediated ER membrane fusion revealed by a FRET-based single-vesicle fusion assay

**DOI:** 10.1038/s41598-017-09162-9

**Published:** 2017-08-18

**Authors:** Kyung Tae Kim, Yeojin Moon, Yunsu Jang, Kang Taek Lee, Changwook Lee, Youngsoo Jun, Sanghwa Lee

**Affiliations:** 1 Advanced Photonics Research Institute, Gwangju Institute of Science and Technology, Gwangju, 61005 Republic of Korea; 2 School of Life Sciences, Gwangju Institute of Science and Technology, Gwangju, 61005 Republic of Korea; 3 Cell Logistics Research Center, Gwangju Institute of Science and Technology, Gwangju, 61005 Republic of Korea; 4 Department of Chemistry, Gwangju Institute of Science and Technology, Gwangju, 61005 Republic of Korea; 5 Department of Biological Sciences, School of Life Sciences, Ulsan National Institute of Science and Technology, Ulsan, 44919 Republic of Korea

## Abstract

Homotypic fusion of endoplasmic reticulum membranes is driven by atlastin GTPases; however, the underlying mechanism remains largely unknown. Here, using a FRET-based single-vesicle fusion assay with liposomes bearing the yeast atlastin Sey1p, we investigated the molecular mechanisms of atlastin-mediated membrane tethering and fusion. Although Sey1p-bearing proteoliposomes frequently underwent membrane tethering in a GTP hydrolysis-dependent manner as reported in studies using bulk assays, only a small fraction of the tethered liposomes proceeded to fusion. Strikingly, the rest of the tethered liposomes failed to fuse or dissociate. This stable tethering, however, did not require continued GTP hydrolysis because GTP omission and magnesium chelation did not disrupt tethering. Interestingly, an increased Sey1p density on the membrane markedly accelerated tethering but barely affected the fusion rate of the tethered liposomes, indicating that Sey1p requires additional factors to support efficient fusion *in vivo*. Finally, the assay also revealed that Sey1p-mediated liposome fusion occurs through hemifusion, suggesting the mechanistic conservation between biological membrane fusion events despite the existence of diverse fusogens.

## Introduction

The endoplasmic reticulum (ER) is composed of flat sheet-like cisternae surrounding the nucleus and interconnected tubules that spread throughout the cytoplasm^1, 2^. ER sheets are studded with ribosomes and thereby serve as a major site for protein translocation, while ER tubules form a reticular structure with physical contacts with other organelles, presumably mediating material transfer between organelles^3^. The reticular structure of ER tubules is established and maintained partly through homotypic membrane fusion between ER tubules^4–6^. Homotypic fusion of ER tubules is mediated by atlastins and their functional homologue Sey1p in metazoans and yeasts, respectively^5, 6^. Atlastins belong to a membrane-bound, dynamin-like GTPase family, and are composed of a GTPase domain, a helical bundle domain, two transmembrane domains and a short cytosolic tail that contains an amphipathic helix^7–9^. In the presence of GTP, the GTPase domain of atlastins can form a homo-dimer *in trans*, which can bring two membranes into apposition and initiates membrane tethering^7, 10^. Then, GTP hydrolysis further tightens membrane docking and induces conformational changes of atlastin molecules, which somehow leads to lipid bilayer merger^7, 11–13^. Although previous studies using X-ray crystallography and FRET-based liposome bulk fusion assays^14, 15^ have provided a general idea of how atlastins mediate membrane fusion, the molecular details of the fusion process remain unclear. In the widely used liposome bulk fusion assays^4, 5, 7^, atlastins are reconstituted into two distinct populations of liposomes, in which one population contains both acceptor and donor fluorophore-conjugated phospholipids and the other carries no fluorophores. Upon fusion, lipid mixing between the two populations of liposomes leads to lateral diffusion of the fluorophores along the lipid bilayers and thereby an increase in the distance between donor and acceptor fluorophores. Fusion between the two populations of liposomes can thus be estimated from the recovery of donor fluorescence. Although it can reveal average behaviours of the two liposome populations, this type of liposome fusion assay (also referred to as bulk fusion assay) cannot differentiate different stages of the fusion process, such as tethering, hemifusion and full fusion, due to population averaging inherent in ensemble measurements. To overcome this limitation, in this study, we established a FRET-based single-vesicle fusion assay using liposomes that bear the yeast atlastin Sey1p to monitor the time course of individual reaction steps during Sey1p-mediated ER membrane fusion in real time. Using this assay, we explored the kinetic pathways of Sey1p-mediated fusion and the role of GTP hydrolysis in the fusion process and made the following observations: (1) Sey1p-mediated vesicle tethering strictly relied on GTP hydrolysis; (2) the majority of the vesicles failed to fuse, and only a small fraction of tethered vesicles proceeded to membrane fusion via hemifusion intermediates; and (3) the majority of the vesicles, which failed to fuse, remained tethered for a long time even in the absence of further GTP hydrolysis, indicating that continuous GTP hydrolysis is not required to maintain Sey1p-mediated membrane tethering.

## Results

### A single-vesicle FRET fusion assay for observing Sey1p-mediated ER membrane fusion

**Figure 1:**
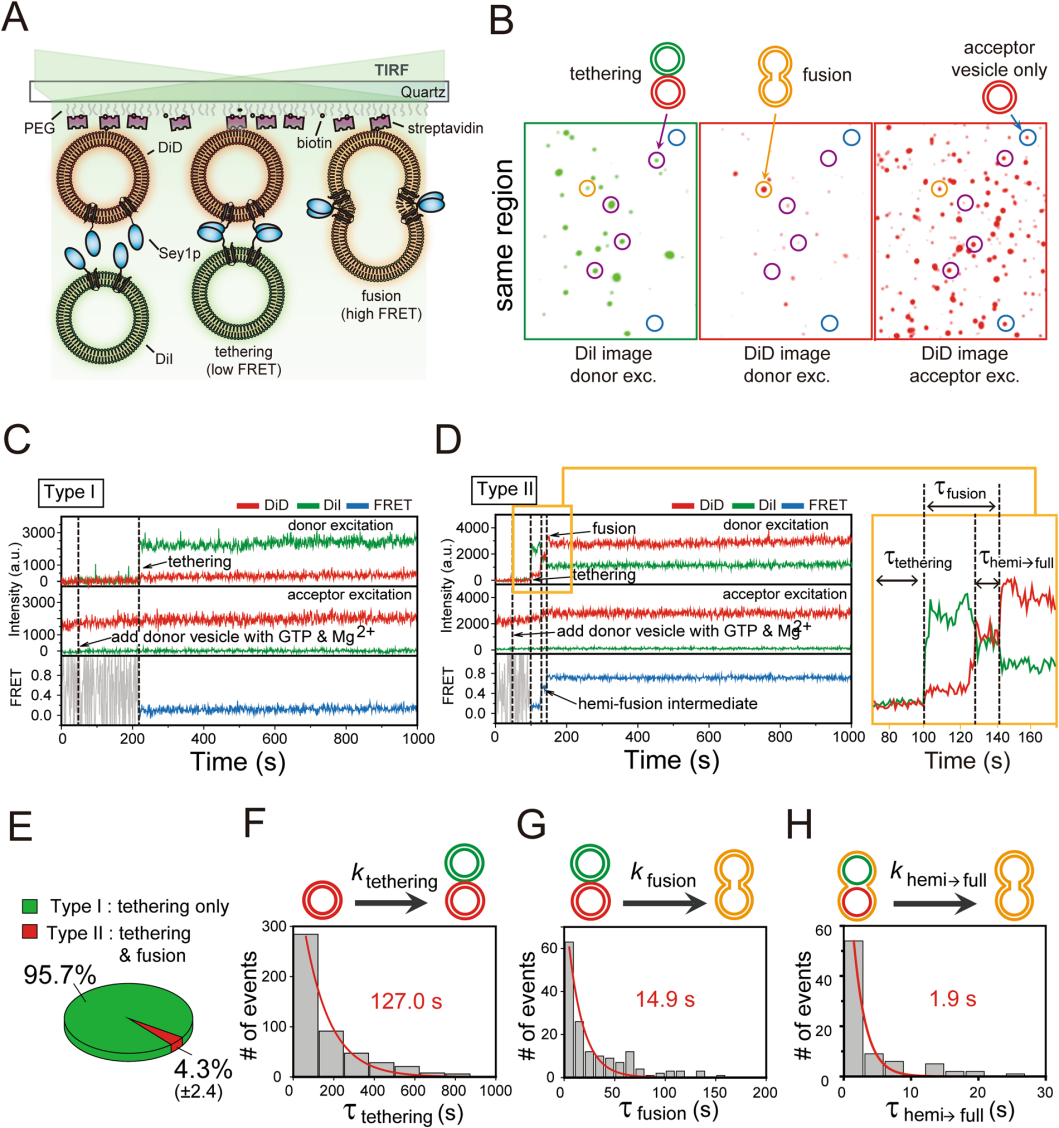
Real-time observation of Sey1p-mediated ER membrane fusion. (**A**) Schematic diagram of the single-vesicle FRET fusion assay. (**B**) Single-vesicle images after the addition of donor vesicles containing Sey1p protein to surface-immobilised acceptor vesicles containing the Sey1p protein upon donor (532 nm laser) and acceptor (637 nm laser) excitation. Tethered and fused vesicles are shown in single-vesicle images. DiI signals are from tethered or fused vesicles under donor excitation (left). DiD signals are from surface-immobilised acceptor vesicles only under acceptor excitation (middle) and from fused vesicles under donor excitation (right). Blue, purple and orange circles indicate where acceptor vesicles only, tethered vesicles and fused vesicles are located, respectively. (**C**,**D**) Representative time traces showing tethering only (**C**) or both tethering and fusion events (**D**) in the presence of 0.5 mM GTP and 1 mM Mg^2+^. DiI fluorescence, DiD fluorescence and the corresponding FRET efficiency are shown in green, red and blue, respectively. The same colour convention is used throughout the paper. (**E**) Fractions of traces showing tethering only and both tethering and fusion events. These fractions were determined by analysing at least 900 tethering events from three independent experiments. (**F**–**H**) Dwell time histograms for individual reaction steps denoted in Fig. 1D, including the tethering (**F**), fusion (**G**) and hemi-to-full fusion (**H**) steps. All histograms were fitted to single-exponential decay functions to obtain kinetic rates. Dwell time histograms for tethering, fusion and hemi-to-full fusion steps were obtained by analysing 481, 159 and 84 events, respectively. Experiments were performed using proteoliposomes with a protein-to-lipid ratio of 1:200.

In the single-vesicle FRET fusion assay (Fig. 1A), we immobilised one group of vesicles (called acceptor vesicles) containing Sey1p proteins and acceptor fluorophores (DiD) on a polyethylene glycol (PEG)-coated imaging surface using biotinylated lipids of the vesicles and monitored fluorescence signals from single vesicles using total-internal-reflection fluorescence (TIRF) microscopy. Then, a second group of vesicles containing Sey1p proteins and donor fluorophores (DiI) were added to a detection chamber while single-vesicle FRET images were being taken to observe vesicle-vesicle interactions in real time. In this assay, we used the alternating-laser excitation technique^16, 17^ to detect both donor-acceptor FRET signals upon donor excitation (532 nm) and acceptor signals upon direct acceptor excitation (637 nm), which enabled us to clearly identify individual reaction steps towards full fusion including vesicle tethering and subsequent membrane fusion. Figure 1B shows typical images of single vesicles upon both donor and acceptor excitation, where each spot represents an acceptor vesicle only (blue circle), a tethered vesicle (purple circle) and a fused vesicle (orange circle). Also, we found that DiI fluorescence spots were observed only in the presence of DiD acceptor vesicles (see purple and orange circles), which indicates that DiI fluorescence appeared as a result of vesicle-vesicle interactions. This conclusion is further supported by the fact that DiI donor vesicles were not observed in the absence of surface-immobilised acceptor vesicles (Supplementary Fig. S1). Furthermore, to test whether the vesicle-vesicle interactions are driven by Sey1p dimerization *in trans* between two fusing membranes, we performed single vesicle FRET fusion experiments using donor vesicles lacking Sey1p proteins, and found that vesicle tethering events were rarely observed (Supplementary Fig. S2).

### Real-time observation of single-vesicle interactions

A major advantage of our single-vesicle FRET fusion assay is the capability to observe single-vesicle fusion dynamics in real time. Figure 1C,D shows the fluorescence intensity and FRET time traces of a representative single vesicle. Tethering of a donor vesicle to a surface-immobilised acceptor vesicle was observed as an abrupt appearance of DiI fluorescence signal, and the fusion of two vesicles was observed as a subsequent increase in FRET efficiency. Interestingly, in the above observations, the interactions after vesicle tethering can be classified into two distinct types (Fig. 1C–E). In most of the tethering events (Fig. 1C; type I, 96%), we did not observe any FRET changes for quite a long time (~15 min) after vesicle tethering, indicating that no fusion occurred. By contrast, we observed a FRET increase immediately after vesicle tethering in the remaining cases (Fig. 1D; type II, 4%). This finding indicates that most of the Sey1p-mediated membrane tethering events did not proceed to membrane fusion. Our single-vesicle observations indicate that the efficiency of fusion by Sey1p alone was markedly low and reflect the requirement for other regulatory factors that trigger fusion after Sey1p-induced membrane tethering. Moreover, our data suggest that the low fusion efficiency of Sey1p was not due to the slow fusion rate. Rather, it was because only a small fraction of tethering events proceeded to the subsequent fusion step. Additionally, in most of the fusion events that we observed, one intermediate FRET state, which most likely represents a hemifusion step towards full fusion, was detected (Fig. 1D). Overall, we conclude that fusion reactions mediated by Sey1p involve three distinct steps: membrane tethering, hemifusion and finally full fusion. From the fitting of the FRET histograms for each state to Gaussian distributions (Supplementary Fig. S3), we observed a FRET distribution centred at 0.08 for the tethering state, 0.35 for the hemifusion state and 0.71 for the full-fusion state. With the 1 s time resolution used in the experiment, a previously uncharacterised sequence of events from tethering to full fusion could be successfully detected in real time, and thus kinetic parameters for individual reaction steps could be determined from the dwell time analysis of each stage (Fig. 1F–H).

### The role of GTP hydrolysis in Sey1p-mediated ER membrane tethering and fusion

**Figure 2:**
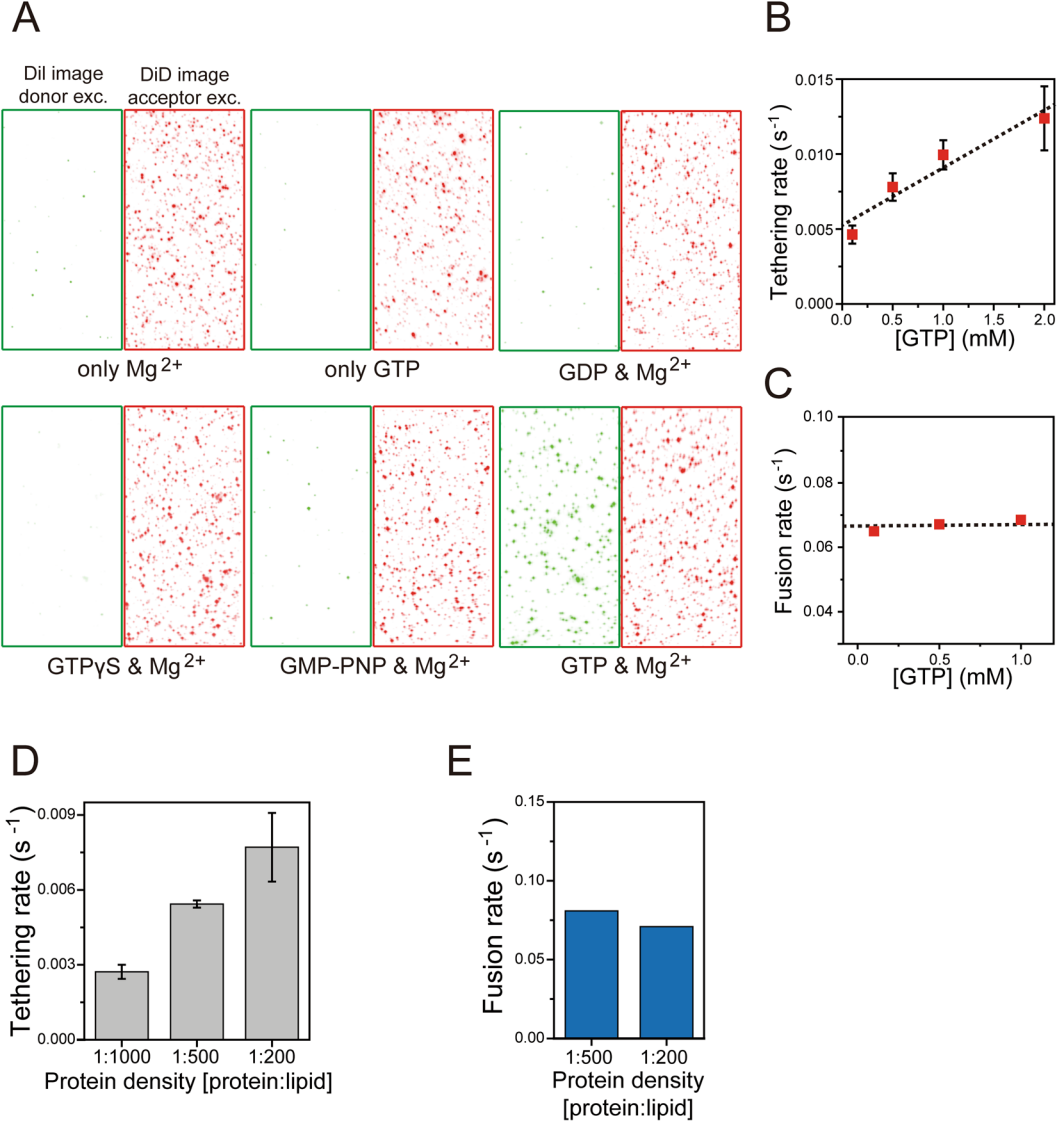
Reaction pathway of GTP hydrolysis in Sey1p-mediated vesicle tethering and fusion. (**A**) Single-vesicle images upon donor and acceptor excitation in various buffer conditions (1 mM Mg^2+^ only, 1 mM GTP only, 1 mM GDP with 2 mM Mg^2+^, 1 mM GTPγS with 2 mM Mg^2+^, 1 mM GMP-PNP with 2 mM Mg^2+^, and 1 mM GTP with 2 mM Mg^2+^). (**B,C**) Tethering (**B**) and fusion (**C**) rates at varying GTP concentrations. The Mg^2+^ concentration was 2-fold higher than the GTP concentration in the GTP titration experiments. To clearly visualise the correlation of tethering or fusion rates with GTP concentrations, a linear fit line for the tethering rate and the average value of the entire data set for the fusion rate were added. In (**B**), error bars represent standard deviations obtained from two or three independent experiments. (**D,E**) Comparison of tethering (**D**) and fusion (**E**) rates at varying protein densities on the vesicle surface. The protein density on the membrane surface was represented as the protein-to-lipid ratio. Error bars represent standard deviations obtained from at least three independent experiments (**D**). Each data point was generated by dwell time analysis of at least 100 fusion events obtained from several independent experiments (**C,E**). Experiments were performed using proteoliposomes with a protein-to-lipid ratio of 1:200, unless otherwise indicated.

Taking advantage of the unique capability of the single-vesicle FRET fusion assay to identify individual reaction steps, we explored the reaction pathway of GTP hydrolysis in Sey1p-mediated membrane tethering and fusion. Previous studies suggested that GTP hydrolysis is required to induce Sey1p-mediated membrane fusion^5, 7, 11^. However, it remains poorly understood when and how GTP hydrolysis executes its function during the fusion process. To address these questions, we first compared the amount of tethered vesicles from single-vesicle images under various reaction conditions (Fig. 2A). Vesicle tethering occurred only in the presence of both GTP and Mg^2+^ (Fig. 2A). The non-hydrolysable GTP analogues GTPγS and GMP-PNP did not support vesicle tethering in the presence of Mg^2+^. Thus, these results confirmed that GTP hydrolysis is required for the membrane tethering step, consistent with previous results^12, 13^. In addition, increasing concentrations of GTP markedly increased the rate of vesicle tethering, but barely influenced the subsequent membrane fusion rate (Fig. 2B,C; Fig. S4), suggesting that GTP hydrolysis-driven membrane tethering is a rate-limiting step governing the overall rate of Sey1p-mediated membrane fusion reaction. This idea is further supported by the finding that an increased Sey1p density on the membrane accelerated tethering, but not subsequent fusion (Fig. 2D,E; Figs S5–S6).

### Continued GTP hydrolysis is unnecessary to maintain vesicle tethering

**Figure 3:**
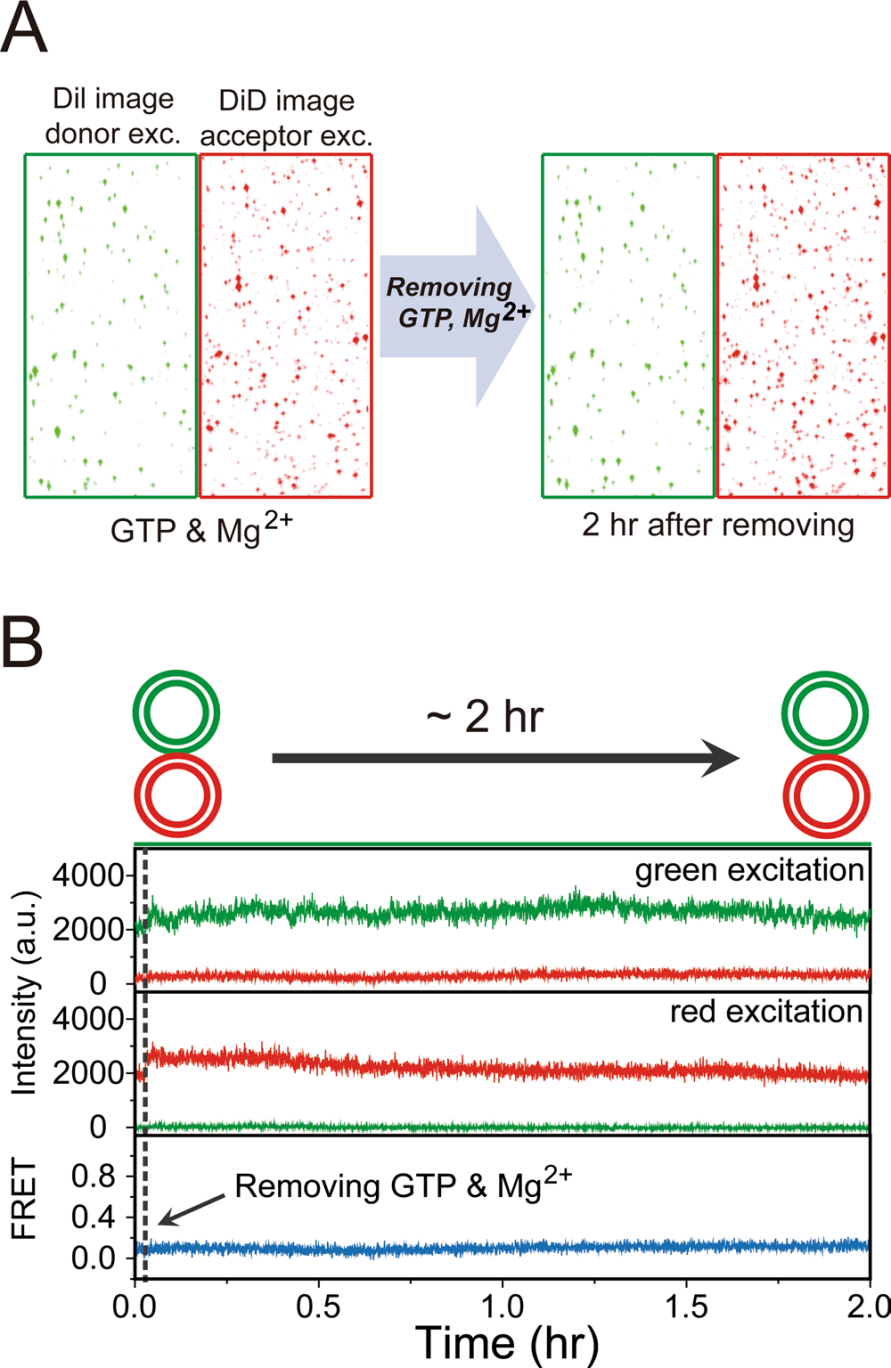
Multiple GTPase cycles are not required to maintain membrane tethering. (**A**) Single-vesicle images upon donor and acceptor excitation before and after removing GTP and Mg^2+^. Three independent experiments all indicated that the tethered vesicles did not dissociate. (**B**) Representative time traces observed in a buffer-exchange experiment. Buffer lacking GTP and Mg^2+^ was injected into the detection chamber during incubation of donor vesicles in the presence of GTP and Mg^2+^. Experiments were performed using proteoliposomes with a protein-to-lipid ratio of 1:200.

A recent study using liposomes bearing human atlastins suggests that continued GTP hydrolysis is required to maintain atlastin-mediated liposome tethering based on the observation that atlastin-induced liposome tethering can be readily reversed by addition of EDTA^12^. To test whether this is the case in our single-vesicle FRET fusion assay, we monitored tethered vesicles for up to 2 hr after removing GTP and Mg^2+^ and then adding EDTA via buffer exchange. Strikingly, the tethered vesicles did not dissociate for 2 hr in the absence of GTP and Mg^2+^ (Fig. 3A,B), strongly suggesting that continued GTP hydrolysis is not required to maintain Sey1p-induced vesicle tethering.

## Discussion

FRET-based single vesicle-vesicle lipid-mixing assays using proteoliposomes have been widely used to study SNARE-mediated membrane fusion and greatly contributed to our understanding of how SNARE proteins and other regulatory factors co-operate to support membrane fusion^18–20^. Our approach described here is the very first attempt to dissect the molecular mechanisms of atlastin-mediated membrane fusion using a FRET-based single-vesicle fusion assay. Real-time monitoring of Sey1p-mediated single liposome fusion revealed several key features: (1) Sey1p mediates efficient liposome tethering in a GTP hydrolysis-dependent manner, but only a small fraction of the tethered Sey1p-bearing liposomes proceeds to fusion; (2) Sey1p-mediated liposome tethering is maintained for a long time even in the absence of continuous GTP hydrolysis; (3) an increased Sey1p density on the liposome accelerates liposome tethering, but not fusion; and (4) Sey1p-mediated liposome fusion occurs via hemifusion.

In single-vesicle lipid-mixing assays with an experimental setting similar to that used in our study^20^, it was reported that donor vesicles bound to accepter vesicles proceed to lipid mixing with a probability of about 20% when both vesicles bear SNARE proteins alone. Interestingly, however, the addition of a single regulatory protein, such as synaptotagmin, to liposomes bearing only SNAREs markedly increases the occurrence of fusion (up to about 60%). Considering the extremely low fusion probability of Sey1p-bearing liposomes (~5%), Sey1p likely needs regulatory proteins to support efficient ER fusion *in vivo*. Consistent with this idea, we recently reported that ER SNAREs co-operate with Sey1p to support efficient ER membrane fusion^21^.

In previous studies using a visual assay for atlastin-mediated tethering and fusion^9, 12^, where one population of atlastin- or Sey1p-containing liposomes are labelled with a red fluorescent dye and the other contains a green fluorescent dye, GTP-dependent tethering or fusion between these two populations is observed as yellow punctae under a fluorescence microscope. Interestingly, these large punctae disappear or become smaller when EDTA is added, indicating that maintenance of tethering requires ongoing GTP hydrolysis. By contrast, our assay revealed that, once vesicles were tethered, their Sey1p-mediated tethering was not disrupted in the absence of GTP and Mg^2+^ or even in the presence of EDTA, suggesting that ongoing GTP hydrolysis is unnecessary to maintain tethered vesicles. This discrepancy may be derived from different experimental approaches, but should be re-addressed by an independent experiment.

Although atlastins and Sey1p do not share sequence homology, they have an identical membrane topology, a similar domain structure/composition and conserved key motifs in the GTPase domain, except that the helical domain between the GTPase domain and transmembrane domains of Sey1p is markedly longer than that of atlastins^14^. Therefore, it has been thought that Sey1p mediates membrane fusion in a similar manner to atlastins. This idea was strongly supported by the observations that metazoan atlastins can replace Sey1p in yeast, and vice versa^4, 9^. Furthermore, a recent study, based on crystal structures of *Candida albican* Sey1p combined with a series of biochemical data, showed that Sey1p functions similarly to atlastins; however, Sey1p forms the tightest dimer in its GTP-bound state, rather than in the transition state during GTP hydrolysis as atlastins do^9^. A similar FRET-based single-vesicle fusion analysis using atlastin-containing liposomes will provide a better understanding of the differences, if any, in the molecular mechanisms underlying membrane fusion mediated by atlastins and Sey1p.

Membrane tethering/docking is a pre-requisite for membrane fusion, and thus specialised proteins mediating membrane tethering (tethering factors) generally co-operate with proteins that drive membrane fusion (fusogens)^22–24^. In SNARE-mediated membrane fusion, for instance, Rab GTPases and their effectors, often called tethering complexes, mediate membrane tethering prior to membrane fusion. Then, SNARE proteins function as a fusogen to complete lipid bilayer merger. In atlastin-mediated ER membrane fusion, however, proteins dedicated to membrane tethering have not yet been identified. Instead, atlastins seem to function in both membrane tethering and fusion. If this is the case, enhancing the activity of atlastins by increasing the GTP concentration or the density of atlastins on liposomes would likely accelerate membrane tethering as well as fusion; however, these modulations only affected the membrane tethering rate, not the fusion rate, in our assay. Thus, these data, together with the extremely low occurrence of Sey1p-mediated fusion, indicate that the major role of Sey1p is to mediate membrane tethering. This is consistent with our recent report that ER SNARE proteins support Sey1p-mediated ER membrane fusion *in vivo* ^21^.

SNARE-mediated intracellular membrane fusion and viral fusion protein-mediated membrane fusion occur via hemifusion intermediates^18, 25–28^, where lipid mixing occurs between two fusing membranes while their luminal contents remain separate, to reduce the amount of energy required for lipid bilayer merger^28^. Time traces of single liposome fusion clearly reveal the existence of hemifusion intermediates during Sey1p-mediated membrane fusion. Although it remains to be determined whether atlastin-mediated membrane fusion occurs through hemifusion intermediates *in vivo*, our data suggest that the molecular mechanisms of biological membrane fusion are well conserved even though different membrane fusion events employ distinct fusion proteins^29^.

## Methods

### Purification of His-Sey1p protein

*Escherichia coli* Rosetta-2 DE3 cells (Novagen) bearing pET-30-*SEY1* (His-3C-Sey1p) were grown in 2 L of LB containing ampicillin (100 μg/ml) and chloramphenicol (37 μg/ml) to an OD600 of 1.0–1.2 at 37 °C. Cells were then incubated at 4 °C for 2–6 hr. Fusion protein expression was induced by treatment with 1 mM IPTG at 16 °C for 16 hr. After obtaining the cell pellet by centrifugation at 5,000 rpm for 5 min at 4 °C, it was resuspended in 40 ml of ice-cold buffer A (20 mM Na-phosphate, 0.5 M NaCl, 10% glycerol, 1 mM EDTA, 1 mM DTT and one tablet of a mini-cocktail protease inhibitor). The mixture was then frozen in liquid N_2_ and thawed in a water bath at 30 °C. Thereafter, 10 ml of Buffer B (20 mM Na-phosphate, 0.5 M NaCl, 10% glycerol, 10% (v/v) Triton X-100 and 1 mM EDTA) was added and incubated at 4 °C for 30 min with gentle agitation. Cells were lysed by sonication, and the lysed cells were centrifuged at 30,000 rpm for 75 min at 4 °C (70Ti rotor; Beckman Coulter). The supernatant was incubated with 1 ml of pre-washed Ni-NTA beads (GE Healthcare) for 4 hr at 4 °C. His-Sey1p-bound beads were washed with 50 ml of buffer C (20 mM Na-phosphate, 0.5 M NaCl, 10% glycerol and 0.4% (v/v) Triton X-100) followed by 50 ml of buffer C containing 20 mM imidazole. His-Sey1p was then eluted with buffer C containing 500 mM imidazole. The eluted His-Sey1p was dialyzed in 2 L of RB150 buffer (20 mM HEPES-NaOH, pH 7.4, 150 mM NaCl and 10% [v/v] glycerol) containing 1 mM EDTA and 0.4% (v/v) Triton X-100.

### Preparation of Sey1p reconstituted vesicles

Reconstitution of proteoliposomes bearing purified recombinant Sey1p was performed as described previously with modifications^4, 5, 21^. All non-fluorescent lipids and biotinyl-PE were purchased from Avanti Polar Lipids except for ergosterol, which was purchased from Sigma-Aldrich. DiI and DiD lipophilic stains were from Molecular Probes. ER-mimicking lipid mixes for His-Sey1p liposomes contained 1-palmitoyl-2-oleoyl-PC (45% or 44.5% [mol/mol] for donor and acceptor His-Sey1p proteoliposomes, respectively), POPE (20%), Soy-PI (10%), DOPS (8.0%), POPA (3.0%), ergosterol (10%), cardiolipin (1.0%), diacylglycerol (1.0%) and lipophilic dyes (2% each of DiI and DiD for donor and acceptor liposomes, respectively), as well as Cap-biotinyl DPPE (0.5%) for acceptor liposomes only. Chloroform was fully evaporated using a stream of N_2_ gas. Dried lipid films of donor or acceptor liposomes were dissolved in RB150 buffer containing 1 mM EDTA and incubated at 37 °C for 1 hr in a shaker. Large unilamellar vesicles were formed by five freeze-thaw cycles using liquid N_2_ and a water bath at 37 °C, and then extruded 11 times through a polycarbonate filter (Avanti Polar Lipids) with a pore size of 100 nm to form unilamellar vesicles. His-Sey1p reconstitution was performed by detergent-assisted insertions as previously described^30^. Purified His-Sey1p in 0.1% Triton X-100 was incubated with preformed unilamellar liposomes at various molar ratios and an effective detergent-to-lipid ratio (R_eff_) of 0.387 at 4 °C for 1.5 hr with gentle agitation. R_eff_ is defined by the following equation: R_eff_ = (D_Total_ − D_water_)/[total lipid], where D_Total_ is the final detergent concentration and D_water_ is the aqueous monomeric detergent concentration (0.18 mM for Triton X-100). After incubation, detergent in the solution was removed by incubation twice with BioBeads SM-2 adsorbent beads (Bio-Rad), which were equilibrated with RB150 buffer containing 1 mM EDTA, for 3 hr and then for 16 hr at 4 °C. Beads were removed from the solution, and the final lipid concentration of proteoliposomes was estimated to be 3.8 mM. To determine the size of vesicles and their homogeneity, dynamic light scattering (DLS) was performed and single-vesicle fluorescence was analysed. The DLS data indicated that the mean diameter of vesicles was about 137 nm. The single-vesicle fluorescence intensity distribution showed that vesicles were mostly populated near the main intensity peak.

### Single-vesicle FRET measurements

To prevent nonspecific adsorption of vesicles, the quartz slide and coverslip were thoroughly cleaned and coated with PEG (m-PEG-5000; Laysan Bio Inc.) and biotinylated PEG (biotin-PEG-5000; Laysan Bio, Inc.) at a 40:1 ratio^31^. Acceptor vesicles containing biotinylated lipids were immobilised on the PEG-coated surface by incubation for 3 min via a streptavidin-biotin interaction. Donor vesicles at a final concentration of 200 pM were injected into the flow chamber to induce vesicle tethering and fusion on the surface. Single-vesicle fluorescence images were taken in a home-built prism-type TIRF microscope with a time resolution of 0.5 s^31^. All measurements were performed using proteoliposomes with a protein-to-lipid ratio of 1:200 in the following buffer unless stated otherwise: 25 mM HEPES, 1 mM GTP and 2 mM MgCl_2._ DiI was excited with a green laser (532 nm, Sapphire; Coherent) for two-colour FRET. Alternative excitation of DiI and DiD was performed with a green laser and a red laser (637 nm, Obis; Coherent) using a mechanical shutter (LS3; Uniblitz). Direct excitation of DiD confirmed the existence of an acceptor vesicle in FRET measurements. Fluorescence signals from DiI and DiD were collected using a water immersion objective lens (UPlanSApo 60×; Olympus), filtered through a 540 nm long-pass filter (LP03–532RU-25; Semrock), separated with a dichroic mirror (635dcxr; Chroma) and imaged onto an electron-multiplying charge-coupled device camera (Ixon Ultra DU897U; Andor). For stable long-term FRET measurement in some experiments, a home-built autofocusing system was employed^32^. During the buffer-exchange experiments, new buffer was infused in real time into the detection chamber using a syringe pump (Fusion 200; Chemyx) while single-vesicle images were being taken. To detect only single-vesicle interactions from heterogeneous populations, a few hundred single-vesicle spots from the images obtained in the acceptor detection channel were selected following 637 nm laser excitation. Individual fluorescence time traces were extracted from a recorded movie file using IDL software and analysed using a custom program written in Matlab (Mathworks). To calculate the FRET efficiency, which is defined as the ratio of acceptor intensity to the sum of donor and acceptor intensities, data correction was performed for background noise, bleed-through of the donor signal to the acceptor channel and direct excitation of the acceptor fluorophore. To eliminate the effect of size differences between donor and acceptor vesicles on FRET values, only vesicles that showed a uniform fluorescence intensity based on the single-vesicle fluorescence intensity distribution were selected and analysed. Individual reaction steps were determined by the threshold method using fluorescence intensity and FRET time traces. Dwell time histograms of each state were fitted by an exponential decay function to obtain the corresponding kinetic rates (Supplementary Figs S4–S5).

## Electronic supplementary material

Supplementary Figures and figure legends
