# Supplementary material for: Molecular mechanisms of atlastin-mediated ER membrane fusion revealed by a FRET-based single-vesicle fusion assay
Source: Sci Rep. 2017 Aug 18;7:8700. doi: 10.1038/s41598-017-09162-9 (PMC5562884; doi:10.1038/s41598-017-09162-9)
Supplement: Supplementary file 1 — Supplementary Figures and figure legends [file 41598_2017_9162_MOESM1_ESM.pdf]

Supplementary Information

Molecular mechanisms of atlastin-mediated ER membrane fusion revealed by a FRET-based single-vesicle fusion assay

Kyung Tae Kim[1,3,4,#], Yeojin Moon[2,3,#], Yunsu Jang[1,3], Kang Taek Lee[4], Changwook Lee[5], Youngsoo Jun[2,3,*] and Sanghwa Lee[1,3,*]

[1]Advanced Photonics Research Institute; [2]School of Life Sciences; [3]Cell Logistics Research Center; [4]Department of Chemistry, Gwangju Institute of Science and Technology, Gwangju, 61005 Republic of Korea. [5]Department of Biological Sciences, School of Life Sciences, Ulsan National Institute of Science and Technology, Ulsan 44919, Republic of Korea.

[#]These authors contributed equally to this work.

[*]Correspondence and requests for materials should be addressed to Youngsoo Jun (email: junys@gist.ac.kr) or Sanghwa Lee (email: sanglee@gist.ac.kr)

Supplementary Figure Legends

Figure S1. Vesicle-tethering events are not observed in the absence of surface-immobilised acceptor vesicles. Single-vesicle images were acquired upon donor (532 nm laser) and acceptor (637 nm laser) excitation 10 min after the addition of donor vesicles to the streptavidin-uncoated surface. DiI fluorescence was not observed in the absence of acceptor vesicles.

Figure S2. Single-vesicle images obtained 15 min after the addition of donor vesicles lacking Sey1p proteins. Single-vesicle images were acquired upon donor (532 nm laser) and acceptor (637 nm laser) excitation 15 min after the addition of donor vesicles lacking Sey1p proteins to the acceptor vesicle-immobilised surface.

Figure S3. Comparison of FRET histograms for fusion intermediates. (A–C) FRET histograms of tethering (A), hemifusion (B) and full-fusion (C) states. FRET histograms were generated from more than 200 events by collecting FRET values of each state. All histograms were fitted to Gaussian functions to obtain FRET values for fusion intermediate states.

Figure S4. Tethering and fusion times at varying GTP concentrations. (A) Tethering time histograms at varying GTP concentrations. To obtain error bars in Figure 2B, each experiment was repeated two or three times. (B) Fusion time histograms at varying GTP concentrations. Each histogram was fitted by a single-exponential decay function to obtain the corresponding kinetic rate. Experiments were performed using proteoliposomes with a protein-to-lipid ratio of 1:200.

Figure S5. Tethering and fusion times at varying densities of Sey1p on the membrane. (A) Tethering time histograms at varying Sey1p-to-lipid ratios of the vesicles. To obtain error bars in Figure 2D, each experiment was repeated three times. (B) Fusion time histograms at varying Sey1p-to-lipid ratios of the vesicles. Each histogram was fitted by a single-exponential decay function to obtain the corresponding kinetic rate. Experiments were performed with 1 mM GTP and 2 mM Mg^{2+}.

Figure S6. Comparison of fusion efficiencies at varying protein densities on the vesicle surface. The protein density on the membrane surface is represented by the protein-to-lipid

ratio. Error bars represent standard deviations obtained from at least three independent experiments. The fusion efficiency was determined by the fraction of traces showing fusion events. Each fraction was calculated based on at least 300 tethering events.

Figure S7. The size of vesicles and their homogeneity were determined by dynamic light scattering and single-vesicle fluorescence measurement. (A) Dynamic light scattering data showing the mean diameter of vesicles. (B) The fluorescence intensity distribution of single vesicles immobilised on the surface. Six hundred vesicles were analysed to determine the intensity distribution.

Figure S1

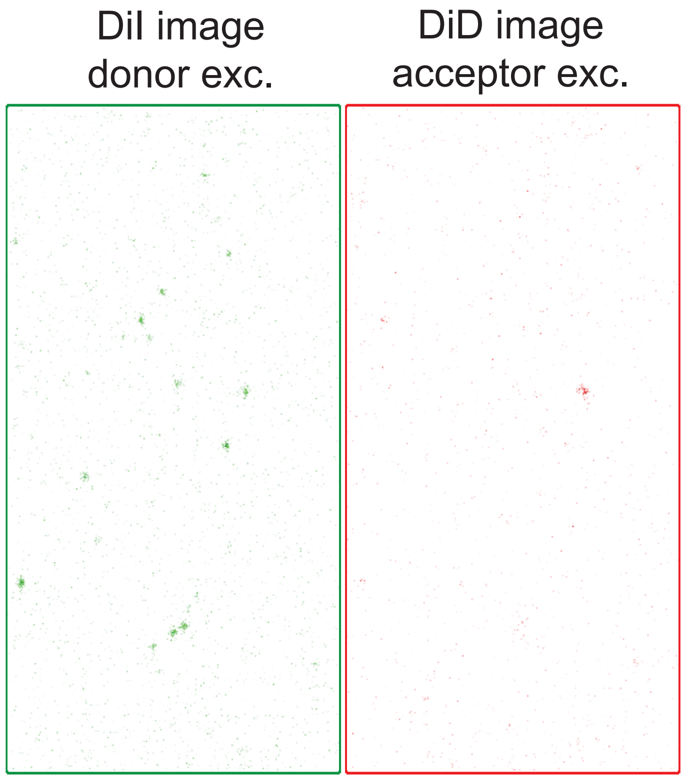

Addition of DiI vesicles to the surface in the absence of acceptor vesicles

Figure S2

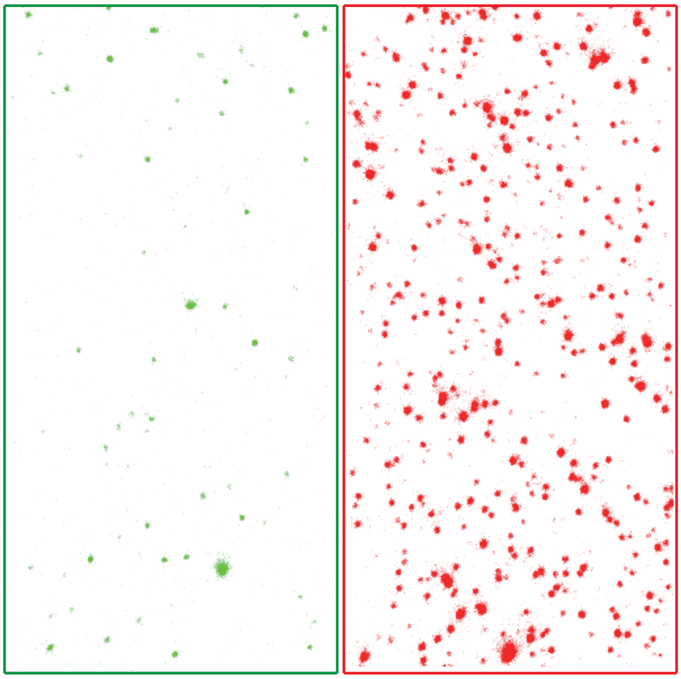

Addition of DiI vesicles lacking Sey1p

Figure S3

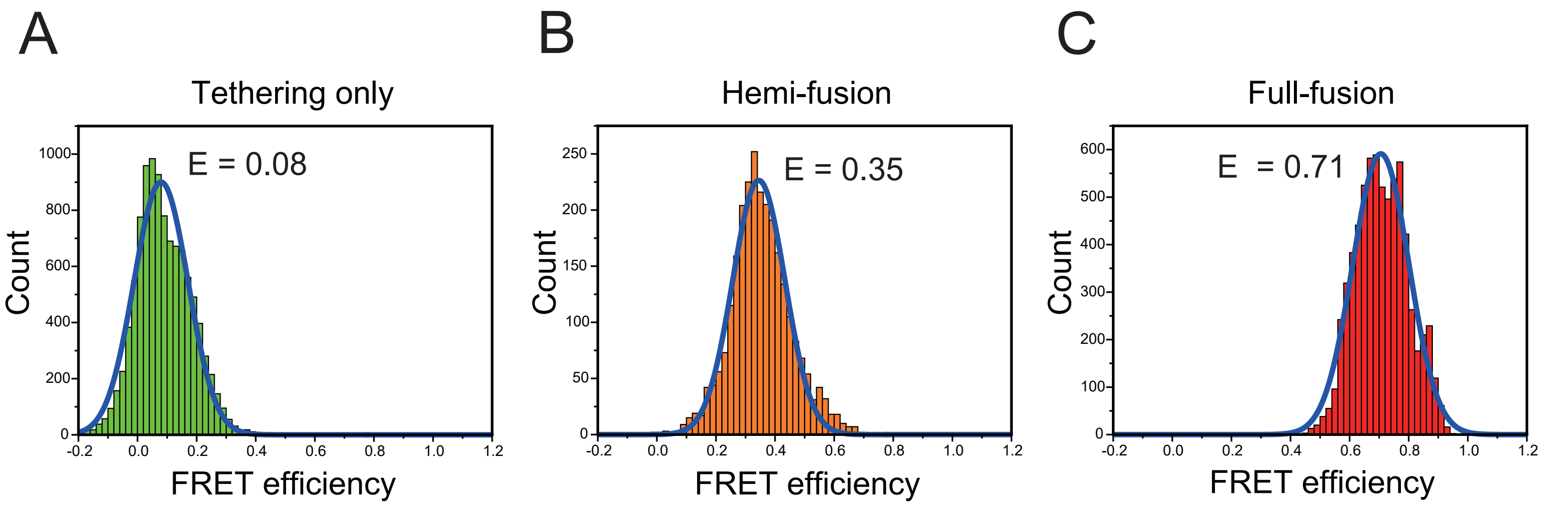
A
Tethering only
E = 0.08
Count
FRET efficiency
B
Hemi-fusion
E = 0.35
Count
FRET efficiency
C
Full-fusion
E = 0.71
Count
FRET efficiency

Figure S4

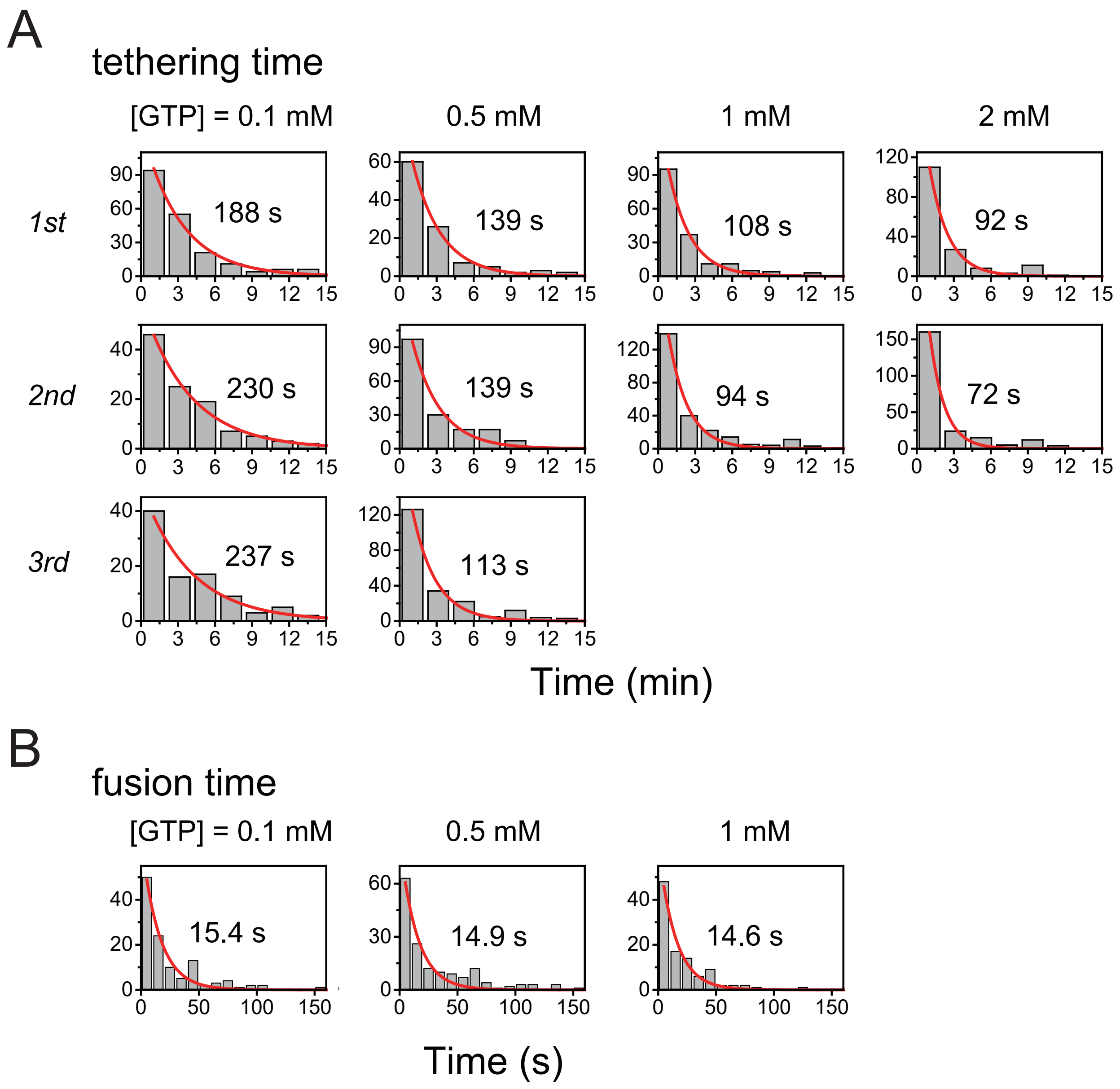

A
tethering time
[GTP] = 0.1 mM
0.5 mM
1 mM
2 mM
1st
2nd
3rd
188 s
139 s
108 s
92 s
230 s
139 s
94 s
72 s
237 s
113 s
Time (min)
B
fusion time
[GTP] = 0.1 mM
0.5 mM
1 mM
15.4 s
14.9 s
14.6 s
Time (s)

Figure S5

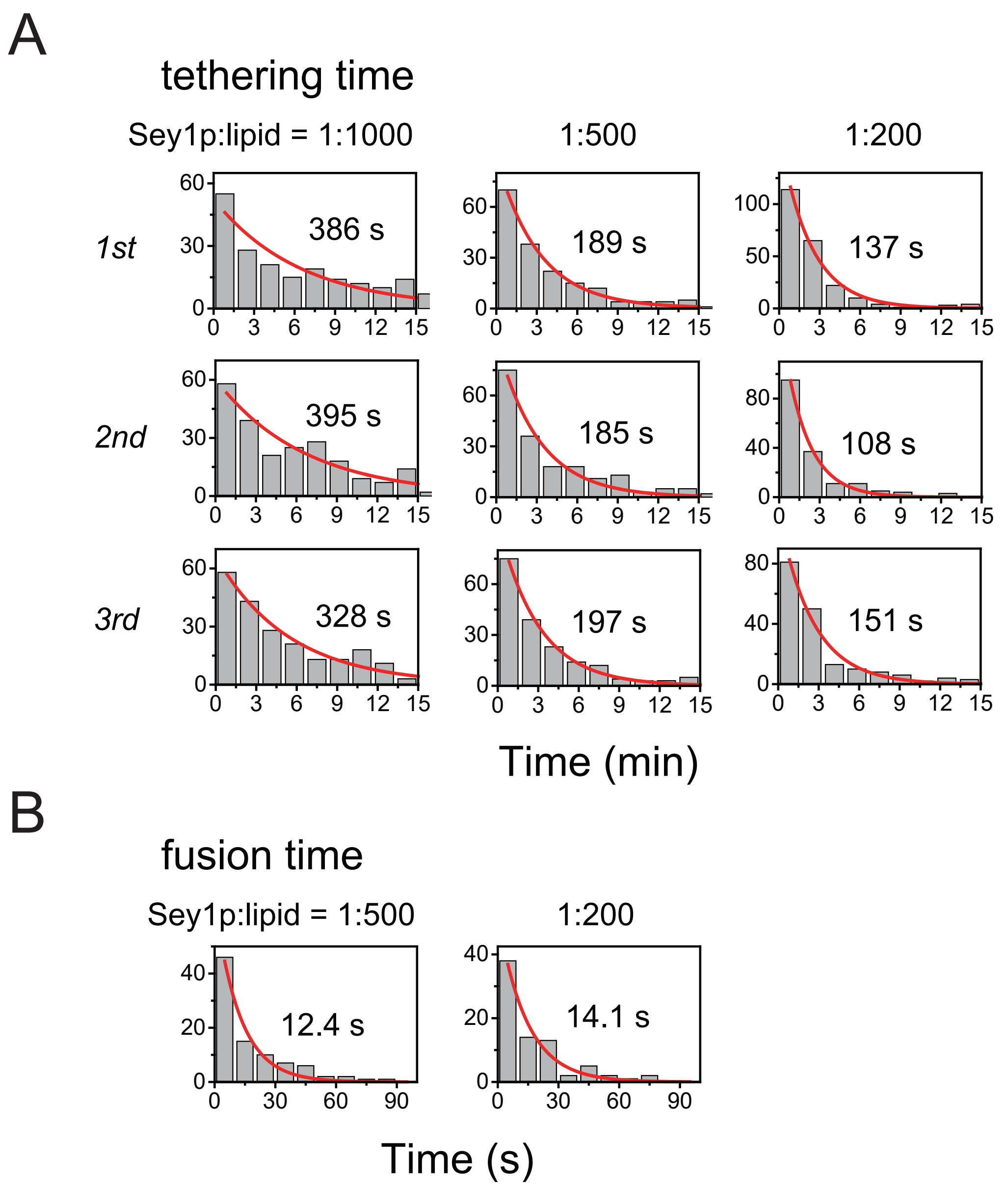
A
tethering time
Sey1p:lipid = 1:1000
1:500
1:200
1st
2nd
3rd
386 s
189 s
137 s
395 s
185 s
108 s
328 s
197 s
151 s
Time (min)
B
fusion time
Sey1p:lipid = 1:500
1:200
12.4 s
14.1 s
Time (s)

Figure S6

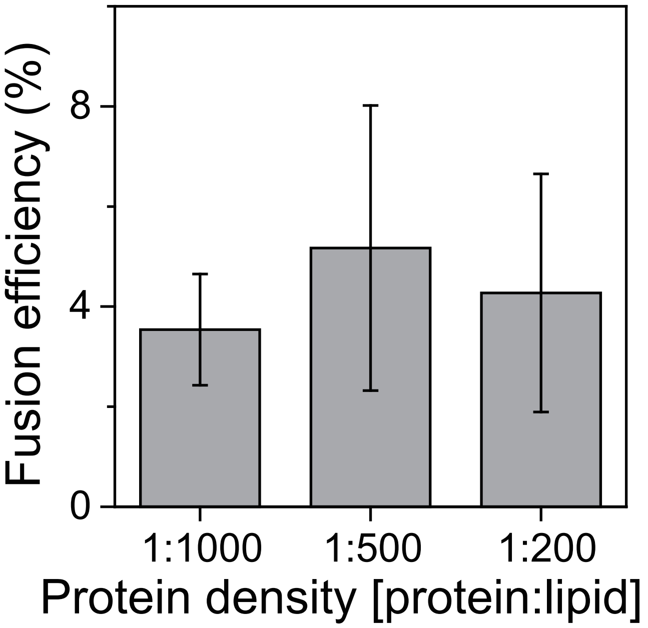

Fusion efficiency (%)
0
4
8
1:1000
1:500
1:200
Protein density [protein:lipid]

Figure S7

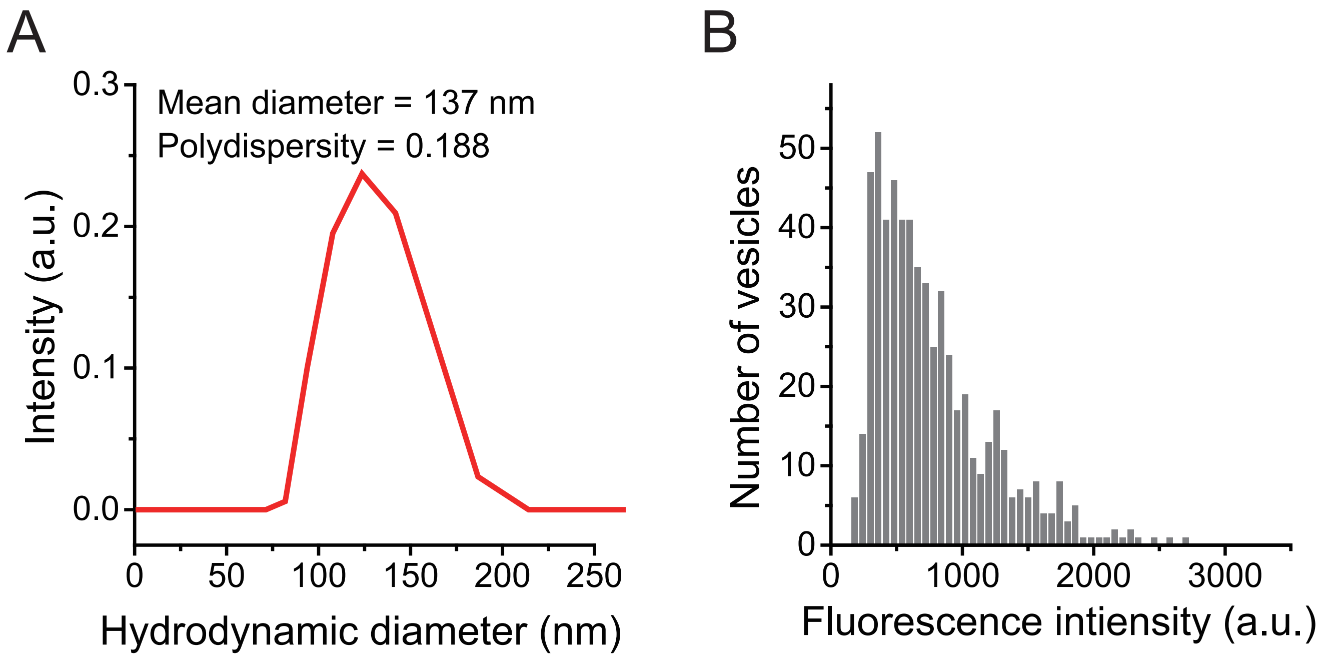
A
B
Mean diameter = 137 nm
Polydispersity = 0.188
Intensity (a.u.)
0.0
0.1
0.2
0.3
0
50
100
150
200
250
Hydrodynamic diameter (nm)
Number of vesicles
0
10
20
30
40
50
0
1000
2000
3000
Fluorescence intiensity (a.u.)
